# Somatization and Body Composition: Findings from a Cross-Sectional Study on Non-Clinical Young Adults

**DOI:** 10.3390/healthcare13030304

**Published:** 2025-02-02

**Authors:** Marius Baranauskas, Ingrida Kupčiūnaitė, Jurgita Lieponienė, Rimantas Stukas

**Affiliations:** 1 Faculty of Biomedical Sciences, State Higher Education Institution Panevėžys College, 35200 Panevėžys, Lithuania; ingrida.kupciunaite@panko.lt (I.K.); jurgita.lieponiene@panko.lt (J.L.); 2 Department of Public Health, Institute of Health Sciences, Faculty of Medicine, Vilnius University, 01513 Vilnius, Lithuania; rimantas.stukas@mf.vu.lt

**Keywords:** body fat percentage, body mass index, CUN-BAE, young adults, mental health, public health, somatization

## Abstract

Background/Objectives: Lifestyle is a significant, common, and easily modifiable factor capable of increasing or reducing the risk of acquiring many diseases. Currently, there is a research gap as too little scientific attention has been focused on exploring the relationship between mental health and nutritional status in various populations. Moreover, the association between body composition and somatization has not been fully disclosed. Therefore, this study aimed to assess the associations of body composition with the symptomatology of somatization in an environmentally vulnerable sample of young adults. Methods: A single cross-sectional study included young non-clinical Lithuanian students (*n* = 1223) aged 21.7 ± 3.9. The body adiposity status of the study participants was estimated using both the body mass index (BMI) and the Body Adiposity Estimator (CUN-BAE) method. Fat-free mass was evaluated via the adjusted fat-free mass index equation (FFMI_adj_). The Patient Health Questionnaire (PHQ-15) was applied to assess the severity of the perceived symptoms of a somatic symptom disorder (SSD). Results: The CUN-BAE was considered to be a better predictor of adiposity than the BMI because 14.7% of females and 6.2% of males were interpreted as obese using the CUN-BAE, while the BMI equation identified participants as having a normal body weight. The highest rates of somatization were found in 18.6% of the cohort. Young adults with higher amounts of body fat mass (β: 0.050, 95% confidence interval (95% CI): 0.013; 0.084, *p* = 0.007) and lower FFMI are prone to a higher risk for developing somatization (β: −0.429, 95% CI: −0.597; −0.260, *p* < 0.001). Conclusions: Our study revealed that body composition is significantly related to multiple somatic complaints throughout a range of measurements. However, in contrast to the CUN-BAE tool, the BMI equation underestimated the relationship between body fat and mental health outcomes in young adults. Even though nutritional status along with targeted physical load, as the mediators, are likely to play a significant role in the maintenance of optimal body composition and mental health outcomes, healthcare providers are recommended to advise individuals to lower their body fat percentage and increase fat-free mass in order to reduce the risk of somatization.

## 1. Introduction

Lifestyle is a significant, common, and easily modifiable factor capable of increasing or reducing the risk of acquiring many diseases. Many research studies have reported the benefits of healthy lifestyle practices for reducing the risk of adverse events [1,2,3]. In this context, proper nutrition is a vital component of human life. Nutrition not only ensures the body’s demands for macronutrients and trace elements but also provides energy for cellular processes in order to maintain homeostasis. However, over the last decades, an increased incidence of obesity leading to the risk of type 2 diabetes, cardiovascular diseases, pulmonary diseases, cancer, and depression has been associated with poor dietary habits that have been described as major triggers [4].

Evidently, many studies have revealed the effects of various dietary regimes on the augmentation of health status and its characteristics; therefore, the Mediterranean and Nordic diets, low-fat and high-protein diets, and diets low in carbohydrates have been identified to play a significant role in protection against the development of non-communicable diseases [5,6,7,8,9,10,11,12].

Nevertheless, currently, there is a research gap as too little scientific attention has been focused on exploring the relationship between mental health and nutritional status in various populations. It should be highlighted that according to the Health Measurement and Evaluation Study, in 2021, 3.8% of the population, or 280 million people worldwide, were affected by depression [13]. Meanwhile, the other most common mental disorder associated with somatic symptoms has been less researched. Somatization is not only a mental health disorder but also a comorbidity for depression [14,15,16] and anxiety [16]. Moreover, it is a common adult condition resulting in functional impairment or increased disability and is a high-cost burden in healthcare [17,18,19]. Somatization is defined as a mental phenomenon related to symptoms of physical origin, i.e., somatic symptoms characterized as “physio-somatic” symptoms likely to lead to distress and/or functional problems [20]). Even though such somatic symptoms affect approximately 10% of the entire population [21,22,23], they tend to receive neither a medical explanation nor are they linked to medical diagnoses or organic causes [18]. In addition, somatic symptoms are less commonly reported by males than females [24]. Moreover, from the perspective of prognosis, an organic explanation of the cause of somatic complaints is not relevant since medical diagnoses for people with somatic symptomatology are the same or even worse than for those with similar symptoms [21,25,26].

Although the above-mentioned non-communicable diseases are related to somatic symptoms [27,28,29], such as perceived chronic stress, fatigue, or pain, the research question is developed on the basis of particular dietary patterns of relatively healthy people who have not yet been diagnosed with non-communicable diseases associated with somatic symptoms and exploring whether dietary modification could benefit from reduced symptomatology of somatization. The latter question is of crucial importance due to the fact that non-specific somatic symptoms have been recognized to serve as prognostic derivatives in the development of non-communicable diseases independent of potential risk factors related to a high body mass index (BMI) or physical inactivity [30,31,32].

Moreover, the human body’s composition is likely to have a significant relationship with mental health outcomes as it is known to be directly influenced not only by eating habits but also by levels of physical activity. Previous research has associated obesity with depression in adult cohorts [33,34] and highlighted that depressive symptoms were 55% more common in obese adults [35]. Furthermore, obesity was recognized as a causal risk factor for the development of depression in a Mendelian randomization study in the UK Biobank [36]. Meanwhile, there are only a few similar research studies on the association between body weight and somatization symptoms. For example, Rush et al. [37] found that military and veteran members with obesity are more likely to screen positive for mental health outcomes, including depression and multiple somatic symptoms.

Additionally, in previous studies, only the body mass index (BMI) has been used as a substitute for body fat estimations based on the assumption that individuals have a sufficiently similar body composition. However, high variability in the core components of body composition (in terms of fat-free mass and body fat mass) is common in people with a similar BMI [38]. Also, the difference in body fat percentage (BF%) was identified to be more significant compared to the difference in BMI, assuming that the use of the BMI equation led to the underestimation of overweight and/or obesity in the target population [39].

Given that the relationship between body composition and somatization has not been fully disclosed, in order to improve the accuracy of scientific findings, our study examined the category of students aged between 18 and 29 years who, due to emerging adulthood, are more susceptible to mental health disorders [40]. In such cases, screenings for mental health issues, including somatic symptoms, should be conducted as soon as possible due to the symptomatology of mental disorders in both adolescents and/or emerging adults likely to lead to more serious mental health outcomes at a later age [41,42,43,44,45,46,47]. This study aimed to assess the associations between body fat and fat-free mass with the symptomatology of somatization in an environmentally vulnerable student-aged population emerging into adulthood. The following research hypotheses (Hs) were constructed:

**H1:**  The severity of somatic symptoms relates to both body mass index and the estimated body fat percentage.

**H2:**  The estimated fat-free mass percentage is associated with the severity of somatic symptoms.

**H3:**  The estimated amounts of body fat mass and fat-free mass are associated with particular somatic complaints.

## 2. Materials and Methods

### 2.1. Study Design and Population

An online-based single cross-sectional study was performed in five of Lithuania’s largest cities, namely Vilnius, Kaunas, Klaipeda, Siauliai, and Panevezys. An a priori representative sample size (*n* = 1059) from an eligible population (*N* = 137,582) with a confidence level of 95% and a marginal error of 3% was calculated from a cohort of non-clinical students via the OpenEpi version 3.01 [48]. The snowball sampling technique was applied during the recruitment procedure of subjects. Young 18–29-year-old non-clinical students enrolled in higher education institutions in Lithuania were invited to participate in the observational study. More specifically, in 2022–2023, the participants were recruited through the websites of 45 official social media groups administrated by seven colleges and six universities in Lithuania. A web-based E-survey research application (Apklausa version 204) was used to collect data from study participants (https://apklausa.lt) (accessed on 15 April 2022). All the students from the general set were given equal access to enter the study sample via E-survey. The present study included all participants classified as (1) Lithuanians without a history of reported mental health disorders; (2) subjects currently studying at a higher-education institution; and (3) participants 18 years of age or older. Secondly, of the eligible population, 136,359 students were eliminated from the study because of exclusion criteria or refusal to participate in the E-survey. The exclusion criteria were defined as follows: (1) students who declined to participate in the study (*n* = 136,295); (2) pregnant women (*n* = 15); and (3) subjects with reported chronic conditions such as diabetes (*n* = 8), hypertension (*n* = 4), bronchial asthma (*n* = 7), gastric ulcer (*n* = 2), medical diagnosis of anxiety and (or) depression (*n* = 27), and cancer (*n* = 1). Finally, over the period of September–November 2023, the data of young non-clinical Lithuanian students (*n* = 1223) 18–29 years of age were included and analyzed. The sample comprised 82.7% females and 17.3% males. According to the branches of science investigated, the subjects were as follows: Medicine and Health Sciences (*n* = 552; 45.1%), Social Sciences (*n* = 378; 30.9%), Natural Sciences and Humanities (*n* = 213; 17.4%), and Technological Sciences (*n* = 80; 6.5%).

### 2.2. Measurements

#### 2.2.1. Psychological Status and Physical Activity

A four-part online confidential E-survey was used. The first part collected the demographic and anthropometric characteristics of participants, including sex (the response options were “male” or “female”), age (in years), a branch of sciences (from the response options “Medicine and Health Sciences”, “Natural Sciences”, “Social Sciences”, “Humanities” or “Technological Sciences”), civil status (the response options were “single”, “divorced”, or “married”), and income level (the response alternatives were “EUR < 300 per month”, “EUR 301–500 per month”, or “EUR > 501 per month”).

The second and third sections of the survey deployed the Patient Health Questionnaire (PHQ-15) [49], the Baecke Physical Activity Questionnaire (BPAQ) [50], and the Hospital Anxiety and Depression Scale (HADS) questionnaire [51,52] to assess the severity of the perceived symptoms of a somatic symptom disorder (SSD), anxiety, and depression and to estimate the levels of physical activity in adults with secondary and higher education degrees. It should be mentioned that PHQ-15 is not ideal as a diagnostic questionnaire, as it alone is insufficient to meet the full diagnostic criteria required for a diagnosis of SSD in line with the Diagnostic and Statistical Manual of Mental Disorders (DSM-V) [20]; however, the PHQ-15 as a screening tool can ideally serve as a continuous measure of the severity of physical somatic symptoms and is capable of establishing the association between somatic complaints and various health outcomes. More specifically, the PHQ-15 [49] is a brief, self-administered scale constructed to assess the severity of 15 common somatic complaints classified into physical symptoms (13 questions) and the symptoms associated with depression (2 questions). Each domain is rated on a 3-point Likert scale, ranging from 0 (“not bothered at all”) to 2 (“bothered a lot”). Generally, the total score of the PHQ-15 scale fluctuates between 0 and 30, where higher scores indicate a greater presence of somatic symptomatology. For the PHQ-15, the total score in the category 15–30 identifies the clinically relevant severity of somatic symptoms. In addition, consistently high scores in a particular domain may indicate significant and problematic areas for study participants that might warrant further assessment, treatment, and follow-up. Further details of the study questionnaires are displayed in Table 1.

#### 2.2.2. Anthropometric Characteristics

Body weight and standing height are metrics typically used in public health studies to estimate BMI. Although it is well documented that a high BMI is a potential risk factor for many adverse health outcomes, as part of the study, students were asked to self-report their current height and body weight via the online tool Apklausa (https://apklausa.lt) (accessed on 15 April 2022). It should be mentioned that self-reported BMI has been considered a limitation in some research [62,63,64]. Alternatively, other scientific research has found that both moderate [65] and large population-based cohorts (*n* = 2643) are represented as systemic error-free depending on self-reported BMI [66]; therefore, BMI calculation from self-reports may serve as a valid measurement for males and females across different sociodemographic groups. In this study, web-collected BMI via a cost-effective, quick, and valid equation was calculated from self-reported data as weight in kilograms (kg) divided by the square of standing height in meters (m^2^) [67,68]:

BMI = body weight (kg)/standing height (m)^2^

Four categories of BMI were applied to estimate body weight in students, as follows: (a) “underweight” (BMI < 18.5 kg/m^2^); (b) “healthy weight” (BMI 18.5–25 kg/m^2^); (3) “overweight” (BMI 25–30 kg/m^2^); and (4) “obese” (BMI ≥ 30.0 kg/m^2^) [69].

BMI is commonly used as a predictor of BF%. Nonetheless, whilst the BMI equation as a tool is useful in epidemiological research, it is highly non-specific for estimating BF% at an individual level [70]. Thus, in this study, the BF% was estimated using an easy-to-use and validated predictive equation which may be applied as an initial screening tool in medical practice and has been published by the Clínica Universidad de Navarra, namely the Clínica Universidad de Navarra—Body Adiposity Estimator (CUN-BAE) [71], as follows:

CUN-BAE (BF%) = −44.988 + (0.503 × Age) + (10.689 × Sex) + (3.172 × BMI) − (0.026 × BMI^2^) + (0.181 × BMI × Sex) − (0.02 × BMI × Age) − (0.005 × BMI^2^ × Sex) + (0.00021 × BMI^2^ × Age)

The participants’ age was expressed in years, and sex was codified as male = 0 and female = 1. It should be highlighted that CUN-BAE, via a newer algorithm, was broadly used to assess body composition in clinical research [71,72,73,74]. CUN-BAE was validated by Bod-Pod using air displacement plethysmography (ADP) as the gold standard method (in terms of the range of error, for the ADP test, this was identified as ± 1 to 2.7% [75]) to determine body composition in adults. In clinical practice, CUN-BAE was useful to identify patients at risk for diabetes and cardiovascular diseases [71,73,76]. The study participants’ body fatness was considered “normal” (BF% ≤ 30% in females and ≤20% in males), “overweight” (BF% ranged from 30.1 to 35% in females and varied from 20.1 to 25% in males), or “obese” (BF% > 35.1% in females and >25.1% in males) [69,71].

The fat-free mass index (FFMI) and adjusted FFMI [77,78,79] were calculated as follows:

FFM = body weight (kg) − (1 − BF%/100) (1)

FFMI = fat-free body mass (FFM) (kg)/standing height (m)^2^ (2)

Adjusted FFMI = FFMI (kg/m^2^) + 6.1 × (1.8 − standing height (m)) (3)

The study participants’ FFMI was rated on a scale as follows: “very low” FFMI (male FFMI < 18 kg/m^2^ and female < 15 kg/m^2^), “average” FFMI (male FFMI corresponding to 18–20 kg/m^2^, and female 15–17 kg/m^2^), “above average” FFMI (male FFMI equivalent 20–22 kg/m^2^, and female 17–18 kg/m^2^), “excellent” FFMI (with male FFMI corresponding to 22–23 kg/m^2^, and female 18–19), and “superior” FFMI (with male FFMI ≥ 23 kg/m^2^ and female ≥ 19 kg/m^2^) [78,79].

### 2.3. Variables and Confounders

Our study assessed the symptomatology of somatization as a dependent variable of interest and body composition as independent. Also, when using the PHQ-15 as a screening tool, false positive outcomes could include the symptoms of both generalized anxiety disorder [15] and depressive disorder [16,80,81]; thus, through data analysis, the constructed regression models for prognostic predictions were adjusted for scores obtained from the HADS questionnaire. Therefore, our study evaluated the symptomatology of both anxiety and depression as confounders. Evidently, somatization often leads to impaired functioning [82,83], and it is related to a decrease in physical activity and deterioration in psychological and social well-being [27,84]. Therefore, the physical activity levels of the study participants were also considered part of the potential confounders. Finally, the variable related to the biological sex of study participants served as a confounder as it has been well-documented by research studies that somatic symptoms are less commonly reported by males than females [24]. In summary, according to the data provided above, body composition as an independent variable was proposed to have an association with somatic complaints, and possible factors were identified as confounders, as follows: (1) biological sex; (2) anxiety symptoms; (3) depressive symptoms; and (4) levels of physical activity.

### 2.4. Statistical Data Analysis

The Statistical Package for the Social Sciences (IBM SPSS Statistics) version 25.0 for Windows (IBM Corp., Armonk, NY, USA) was applied to perform statistical data analysis. The graphical visualization of the study data was performed using SPSS software version 25.0. The Kolmogorov–Smirnov test was used to assess the normality of the study data. Continuous variables were represented as means ± standard deviations (SDs), while categorical variables were represented as numbers, and percentages were shown using the relative frequency tables. The differences and correlations in categorical variables (sex, income level, civil status, and categorized score of PHQ-15) were assessed using Pearson’s χ^2^ test coupled with Cramer’s V (*V*) correlation coefficient. The values of *V* were interpreted as follows: “weak correlation” (0 ≤ |*V*| < 0.2), “moderate correlation” (0.2 ≤ |*V*| < 0.4), and “relatively strong and strong correlation” (|*V*| ≥ 0.4). The differences and correlations between continuous variables (the PHQ-15 score, HADS-Anxiety score, HADS-Depression score, and BPAQ score, including scores for sports, work, leisure-time indexes, and anthropometric characteristics) were explored using Welch’s *t*-test (for unequal variances) combined with Cohen’s d (*d*) effect sizes and Pearson’s correlation coefficient (r). In conformity with Cohen [85], the outcomes were categorized into a “small effect size” (0.2 ≤ |*d*| < 0.5), “medium effect size” (0.5 ≤ |*d*| < 0.8), and “large effect size” (0.8 ≤ |*d*| < 1.3). The r values were suggested to be the following: “negligible correlation” (0 ≤ |r| < 0.3), “low positive correlation” (0.3 ≤ |r| < 0.5), “moderate positive correlation” (0.5 ≤ |r| < 0.7), “high positive correlation” (0.7 ≤ |r| < 0.9), and “very high positive correlation” (0.9 ≤ |r|).

Multiple linear regression models were obtained to assess the association between somatic symptoms (PHQ-15) and the core components of body composition (BF% and FFMI_adj_). In all linear regression models, the confounding variables were sex, the scores of the HADS-Anxiety scale, the HADS-Depression scale, the sports index, and the work index. The coefficient of determination (*R*-Squared (*R*^2^)) was calculated to assess the goodness-of-fit of each linear regression model.

In all statistical tests used for data analysis, a 2-tailed *p*-value ≤ 0.05 was assumed to be statistically significant.

## 3. Results

### 3.1. Sociodemographic Characteristics and Somatization

The sample comprised 1012 (82.7%) females and 211 (17.3%) males. The study participants’ average age was 21.7 ± 3.9 (95% CI: 21.5; 21.9) years. Complete information regarding the sample size and the participants’ characteristics is provided in Table 2.

This study highlighted the proportion of young people with the most serious rates of somatization, as reported by the study participants. Generally, the highest rates of somatization were found in 18.6% of the study participants, while 81.4% of the subjects were considered without relevant somatic symptoms.

Furthermore, a series of tests were conducted to assess the significant differences observed by comparing the participants’ sociodemographic characteristics, physical activity levels, and the symptoms of comorbid disorders triggered by somatization. Statistically significant differences between variables were found. Females (21.1%) with severe symptomatology of somatization had statistically higher average scores than males (6.6%) (*V* = 0.14; *p* < 0.001). Also, the study participants suffering from anxiety and depressive symptoms accounted for higher scores on the PHQ-15 (*d* = −1.0 and *d* = −0.8).

Table 2 shows the results of how study participants were undertaking physical activity. The evaluation of the physical activity in the area of sports revealed that the average index score (2.3 ± 0.6) was lower in individuals suffering from somatic symptoms than in subjects without somatic complaints (*d* = 0.3). whose sports index score was (2.5 ± 0.7). In contrast, higher work index scores (2.6 ± 0.7) were obtained in a study subgroup with higher PHQ-15 scores compared to those (2.5 ± 0.6) in subjects without relevant somatic complaints (*d* = −0.3).

Table 3 shows a deeper analysis of the study data related to somatic symptoms. Considering the general sample, the most severe somatic complaints (column ‘1 + 2’) were reported as follows: “feeling tired or having low energy” (94.2%), “back pain” (70.3%), “headaches” (73.4%), and “trouble sleeping” (72.5%). Meanwhile, the less severe somatic symptoms were “shortness of breath” (20.4%), “pain or problems during sexual intercourse” (19.1%), “pain in arms, legs, or joints” (17.9%), and “fainting spells” (8.1%).

### 3.2. Body Composition

As displayed in Table 4, the means ± SDs, as measures of the central tendency of BMI and BF% for males and females, were 23.2 ± 3.7 kg/m^2^, 17.6 ± 6.4%, and 22.0 ± 3.6 kg/m^2^, 27.7 ± 6.1%, respectively.

**Figure 1:**
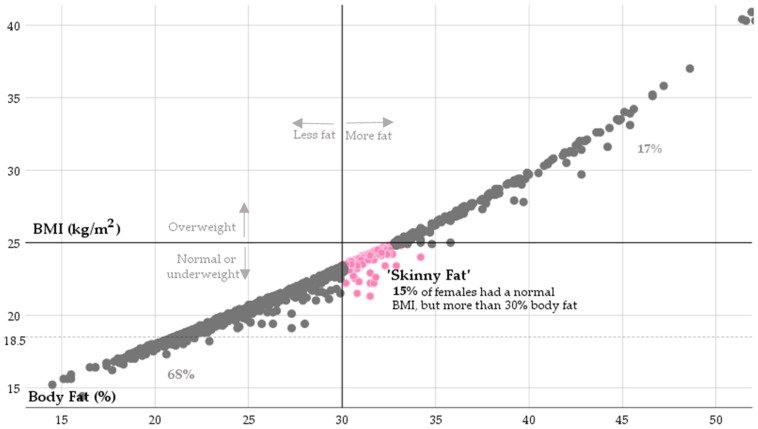
A graphic representation of body weight status according to the outcomes obtained via different methods (BMI vs. CUN-BAE) in a sample of females (*n* = 1012).

Our study revealed a strong correlation (r = 0.77, *p* < 0.001) between BMI and BF%. According to BMI, normal weight was found in 14.7% of females and 6.2% of males, while the use of the CUN-BAE equation revealed higher BF% in obese subjects. Since the BMI equation showed a significant BF% underestimation, specifically in the cohort of females, a more detailed analysis of the adiposity level is represented graphically in Figure 1.

Moreover, whilst BMI incorrectly labeled study participants as being normal, these subjects were labeled as “skinny fat”. Finally, a more in-depth analysis showed that the proportion of obesity in males and females equaled 27% and 17%, respectively.

Additionally, the adjusted fat-free mass index of males and females was assessed as indicated in Table 4. It should be emphasized that the average of the adjusted FFMI in both males (18.7 ± 1.5 kg/m^2^) and females (16.3 ± 1.2 kg/m^2^) met only an “average” limit.

### 3.3. Association Between Body Composition and Somatization

#### 3.3.1. Bivariate Analyses

**Figure 2:**
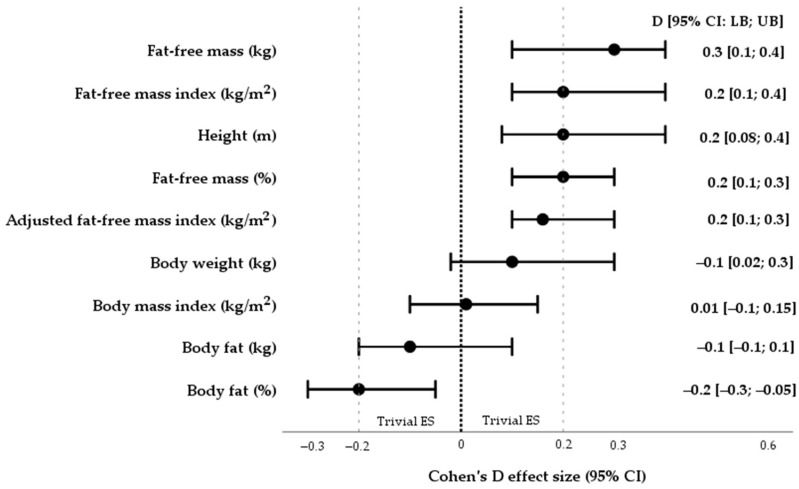
The measure of Cohen’s D reports the effect size of mean differences in the core elements of body composition after comparing them to participants with different symptomatology of somatization (15 ≤ PHQ-15 ≤ 30 vs. PHQ-15 score ≤ 14). ES—effect size; 95% CI—95% confidence interval; LB—lower bound; UB—upper bound.

In the first phase of the association analysis, the statistically significant negative and positive correlations between the somatization and both BF% (r = 0.2, *p* = 0.001) and fat-free mass (r = −0.3, *p* < 0.001) were identified. Additionally, as shown in Figure 2, in terms of effect size (*d*), it can be confirmed that lower values for FFM% and FFMI_adj_ (kg/m^2^) along with lower averages for BF% were established in subjects with severe somatic symptoms (*d* = 0.2 and *d* = −0.2), while BMI (kg/m^2^) outcomes did not differ depending on the symptomatology of somatization (*d* = 0.01).

#### 3.3.2. Regression Analyses

The results of multiple linear regression analyses are presented in Table 5 and Table 6. Multiple linear regressions were constructed to determine how BF% and FFMI_adj_ (kg/m^2^) may predict the total PHQ-15 score as well as the somatic symptoms suffered by the study participants. Two multiple linear regression models were adjusted for confounders, namely the sex, anxiety symptoms, depressive symptoms, and physical activity loaded by subjects. After using the multiple linear regression method, the study revealed the relationship between the dependent and independent variable as follows: (1) a higher BF% was associated with a higher expression of somatic symptoms (β 0.050, 95% CI: 0.013; 0.084, *p* = 0.007); (2) the FFMI_adj_ was associated with the symptomatology of somatization in an adverse way (β −0.429, 95% CI: −0.597; −0.260, *p* < 0.001).

In addition, more in-depth analyses disclosed the association between higher body fat levels and the severity of multiple somatic symptoms such as producing “constipation, loose bowels, or diarrhea”, “nausea, gas or indigestion”, “menstrual cramps”, and “headaches” (Table 5). On the other hand, as shown in Table 6, a lower FFMI_adj_ was associated with a higher number of multiple somatic complaints, namely “back pain”, “thoracic pain in the chest area”, “dizziness”, “feeling tired or having low energy”, “feeling your heart pound or race”, “headaches”, “menstrual cramps”, and “nausea, gas or indigestion”.

## 4. Discussion

This study explored the relationship between body composition and the self-reported severity of somatization among an environmentally vulnerable cohort of adolescents emerging into adulthood. The findings of this cross-sectional study demonstrate that using the BMI equation underestimates the adiposity among young adults with no diagnosed chronic/non-communicable disease attending higher education institutions in Lithuanian cities. Also, the study provides convincing data that somatization is associated with adiposity, as measured by an alternative measure, the CUN-BAE.

### 4.1. Proportion of Somatic Complaints

The study findings revealed a relatively high proportion of severe somatic symptoms suffered by young, non-clinical Lithuanian adults. In the studied sample, a higher proportion rate (18.6%) of severe somatic complaints was found in the sample under analysis compared to 10% reported in the global population [21,22,23]. Moreover, our study identified a higher prevalence rate of somatization compared to the rates reported by other studies conducted in Czech (12.5%) and Slovakia (10.3%) [87], Nigeria (14.3%) [88], and China (7.6%) [89]. The proportion of severe somatic symptoms suffered by young-aged British (31.2%) [90], Arabian (33.8%) [91], Bahranian (46.2%) [92] and Jordanian (55.0%) [93] student populations was twice as high compared to our study data. In addition, our study found that females exhibited a greater extent of somatic complaints than males, as observed in adult populations from Taiwan, Sweden, and Spain [94,95,96]. Notably, according to the findings of our sample, the most severe somatic symptom, namely, “feeling tired”, was consistent with the findings obtained by Goweda et al. [91], while “back pain” and “trouble sleeping” were reported to be the most prevalent somatic complaints in the cohorts of Spanish [96] and Bahranian [92] students, respectively.

### 4.2. Body Mass Index vs. Body Fat Percentage

In terms of the estimation and interpretation of body fat in our sample, the authors declined to use the BMI equation, as in 2023, the American Medical Association (AMA) and Diabetes and Endocrinology Commission published reports along with recommendations calling on medical professionals to carry out a more detailed evaluation of overweight and obesity and demonstrate less reliance on BMI outcomes [45,97,98]. Our study revealed that BMI underestimated adiposity since 14.7% of females and 6.2% of males were interpreted as obese using the CUN-BAE; however, the BMI equation identified them as having a normal body weight. In line with this finding, the BMI equation is limited in identifying clinically relevant somatic complaints in study participants compared to the CUN-BAE tool [71].

Given that BMI is a relatively weak approach in predicting BF% at an individual level [70], the study participants’ body fat mass was assessed using the CUN-BAE as this method was validated via Bod-Pod by applying the gold standard ADP test [71,73]. Thus, by applying the CUN-BAE tool, obesity levels were found among both females (17%) and males (27%) under analysis. In this case, the weight status of young Lithuanian adults remained stable, related to their lower odds for the development of obesity compared to the global codification of trends where 33.9% of younger individuals aged 15–40 years have shown the prevalence of central obesity [99].

### 4.3. Body Composition and Somatization

This study found an association between body composition and the risk of developing somatization in young Lithuanian adults. More specifically, the beneficial association of a lower expression of somatic complaints was more pronounced among the study participants with a lower BF% and a higher FFMI_adj_. The findings derived from our study were consistent with data published by Rush et al. [37], claiming that depression, somatization, and post-traumatic stress disorder are significantly more frequent among obese individuals. It is equally important that our findings are in line with some previous studies [100,101,102,103], which reported that “the whole body fat distribution was related to the increased risk of mental outcomes”. A possible explanation for this is that being overweight and/or obese may contribute to depression or somatization due to the potential metabolic effects likely related to the increased glucocorticoid (cortisol) levels and the hyperactivation of the hypothalamic–pituitary–adrenal (HPA) axis [104]. Also, obesity can result not only in the overexpression of appetite-regulating genes in the hypothalamus but also in the under-expression of genes in the adrenal gland [105]. Furthermore, there is evidence relating to the association between regional body fat distribution (in terms of segmental body fat analyses) and higher levels of cortisol stimulating overactivity in the HPA axis as well as increasing the risk of developing mental disorders [103]. However, it could be speculated that social drivers such as psychosocial distress and adversity in childhood can also lead to the development of eating disorders, which can serve as the mediators between body fat status and somatization during emerging adulthood. Therefore, at the next stages of our research, there is a potential need to construct a multi-faceted framework in order to explain the association between body fat and somatization in a complex way.

Additionally, a Mendelian randomization study [36] proposed that a lower fat-free mass did not serve as a risk factor for mental health. On the contrary, a case–control study conducted by Mutz and Lewis [106] disclosed that in individuals with lifetime depression, fat-free mass was significantly higher compared to healthy subjects. In this context, our study findings are ambiguous as they confirmed the relationship between a more developed body with fat-free mass and a lower expression of multiple somatic symptoms such as “back pain”, “thoracic pain in chest area”, “dizziness”, “feeling tired or having low energy”, “headaches”, “menstrual cramps”, and “nausea, gas or indigestion” in a cohort of young Lithuanian adults. Even though our study revealed that the average FFMI_adj_ in both males (18.7 ± 1.5 kg/m^2^) and females (16.3 ± 1.2 kg/m^2^) was considered as just “average”, body fat-free mass development throughout proper nutrition and exercise could potentially be a merit in lowering the expression of somatic complaints among young adults. Second, behavioral intervention is broadly recognized as a beneficial treatment for mental disorders [107]. For example, a systematic large-scale study identified a number of lifestyle factors, namely physical activity and/or dietary habits, that, if modified, are able to improve mental health [108]. Therefore, in order to prevent the gain of body fat, a significant increase in body fat-free mass in young adults with multiple somatic complaints can be encouraged by enhancing the eccentric contractions of synergetic skeletal muscles [109] coupled with a diet guided in the consumption of low-fat foods and the adoption of a high-protein diet [110].

### 4.4. Strengths and Limitations

In agreement with a relatively large representative sample of young Lithuanian adults and the use of reliable and modern study instruments for data analysis, for the first time, our study assessed the association between body composition and the risk of developing somatization, which is a major strength of our study. The second strength of our study is related to the management of subjects, especially females with somatic complaints [40]. In terms of an extensive form examining mental health disorders such as depression, anxiety, or somatization, this study focused on the early-phase identification of relevant somatic complaints. Therefore, the student-age population can be helped by healthcare professionals to avoid negative mental health outcomes.

However, some limitations of the study need to be reviewed. Even though an absolutely accurate method for assessing BF% does not exist, the CUN-BAE equation referring to the third level of validity approach cannot replace ADP or DXA when performing the assessment of body composition at an individual level. Also, the standing height and weight of study participants were collected in a self-reported mode, increasing the potential risk of biases related to underestimating or overestimating [62,63,64], especially when calculating CUN-BAE via BMI.

Secondly, whilst the studied sample size was representative, the generalizability of the results can potentially be limited by the fact that the sample consisted exclusively of young Lithuanian adults and included more than 80% of females. Moreover, the transferability of study results to other populations can depend on different lifestyle factors (e.g., eating habits, coping strategies for stress management, exercise regimes); therefore, different lifestyles may influence both body composition and somatization. In this case, our study results can be extrapolated to young adult female populations on a larger scale only in Eastern Europe. Finally, this study was cross-sectional in design, which limited the establishment of a cause-and-effect relationship between the variables explored.

## 5. Conclusions

The study highlights a worrying proportion of somatization when considering how almost every fifth young-aged non-clinical adult individual was identified as suffering from severe somatic symptoms. Additionally, our study revealed that body composition is associated with multiple somatic complaints through a range of measurements. More indicatively, young adults with higher amounts of body fat mass and a lower fat-free mass index are related to a higher risk of developing somatization. However, in contrast to the Body Adiposity Estimator, the body mass index equation, due to underestimation, was restricted in detecting the association between body fat and mental health outcomes in young adults. Within this context, future research using longitudinal or experimental designs will undoubtedly be able to validate the results obtained from our study. Furthermore, whilst nutritional status along with a targeted physical load as mediators may play a significant role in the maintenance of optimal body composition and mental health outcomes, it is recommended for healthcare providers to advise individuals to reduce the risk of somatization by lowering their body fat percentage and increasing fat-free mass. Finally, although biological mechanisms, including their role in understanding the association between body composition and somatization, proposed in this study are well-known, exacerbating psychosocial stress or psychological distress attributable to pre-existing risk factors may also be considered as triggers for the development of both obesity and somatization among emerging adulthoods. Therefore, it is feasible to propose future research directions to account for developmental stressors, including childhood adversity and allostatic loads associated with the cumulative burden of chronic psychological stress and life events.

## Figures and Tables

**Table 1:** Characteristics of instruments used in the observational study.

Instrument	Content	Scaling
A Sociodemographic Questionnaire (7 items).	Information about age, sex, civil status, branch of science, income, and anthropometric characteristics.	Nominal scale: 0 = “No” to 1 = “Yes”. Ratio scales.
The Patient Health Questionnaire (PHQ-15) (15 items) [49].	The PHQ-15 is a standardized [53] and moderately reliable (Cronbach’s coefficient alpha α = 0.78) [54,55] questionnaire for the detection of subjects at risk of somatoform disorders. The PHQ-15 helps to measure the severity of perceived somatic symptoms over the past month. The scale PHQ-15 includes 15 common somatic symptoms that account for more than 90% of the physical symptoms reported, excluding upper respiratory symptoms. The PHQ-15 scale includes 2 physical symptoms associated with mental health—“feeling tired or having little energy”, and “trouble sleeping”.	A three-point Likert scale: 0 = “not bothered at all” to 3 = “bothered a lot”. A high score on the PHQ-15 scale is strongly associated with the counts of physician-rated somatoform disorder symptoms [17,56]. There are cut-offs for the PHQ-15 scale. In the PHQ-15, the total scores in the categories 0–4, 5–9, 10–14, and 15–30 identify the severity of somatic symptoms as follows: “none”, “mild”, “moderate” and “severe” [54].
The Baecke Physical Activity Questionnaire (BPAQ) (16 items) [50].	The BPAQ, as a reliable and valid questionnaire, was designed to assess the levels of physical activity in adults [57,58,59,60] with secondary and higher education degrees [61]. The BPAQ helps to evaluate the physical activity index over the last 12 months in three dimensions: (1) work activity; (2) sports activity; and (3) leisure-time activity.	A five-point Likert scale: from 1 to 5. According to the sum of the three domains score, a total physical activity score can range from 3 to 15. The BPAQ allows us to interpret how adults are undertaking physical activity; however, there are no cut-off points for the BPAQ and domains related to the “work index”, “sports index”, and “leisure index” [50].
The Hospital Anxiety and Depression Scale (HADS) questionnaire (14 items) [51,52].	The HADS was constructed to measure the symptoms of depression and anxiety experienced during the previous week. The HADS consists of 2 dimensions: (1) anxiety symptoms (HADS-Anxiety); (2) depressive symptoms (HADS-Depression) [51].	A four-point Likert scale: 0 = “Not at all” to 3 = “Most of the time”. Scores can range from 0 to 3 for each HADS item. The total score for HADS subscales can fluctuate between 0 and 21. There are cut-offs for the HADS subscales. A score of 7 or <indicates asymptomatic anxiety or depression; a score of 8–10 refers to borderline symptomatology of depression or anxiety; a score of 11 and >indicates clinically relevant symptoms of anxiety or depression [51].

**Table 2:** The categorization of individuals with different somatic symptoms.

Variables	Minimal–Moderate Somatic Symptoms (*n* = 996)		High Somatic Symptoms (*n* = 227)		*V* ^a^/*d* ^b^	*p*
	*n*	% or Mean ± SD	*n*	% or Mean ± SD		
Sex						
Male	197	93.4	14	6.6	0.14 ^a^	<0.001
Female	799	79.0	213	21.0		
Income level						
<EUR 300 per month	556	80.1	139	20.0	0.05 ^a^	0.200
EUR 301–500 per month	207	81.5	47	18.5		
EUR > 501 per month	232	85.0	41	15.0		
Civil status						
Single	933	81.1	217	18.9	0.07 ^a^	0.060
Divorced	7	63.8	4	36.4		
Married	56	90.3	6	9.7		
Psychological status						
HADS-Anxiety (score)	9.5 ± 4.4		14.0 ± 3.7		−1.0 ^b^	<0.001
HADS-Depression (score)	5.4 ± 3.5		8.3 ± 3.9		−0.8 ^b^	<0.001
Physical activity						
BPAQ (total score)	7.6 ± 1.3		7.6 ± 1.3		0.1 ^b^	0.424
Sport index (score)	2.5 ± 0.7		2.3 ± 0.6		0.3 ^b^	<0.001
Work index (score)	2.5 ± 0.6		2.6 ± 0.7		−0.3 ^b^	<0.001
Leisure-time index (score)	2.7 ± 0.6		2.7 ± 0.6		0.1 ^b^	0.226

^a^—Cramer’s V correlation coefficient (*V*); ^b^—the effect size (*d*); SD—standard deviation; *p*—*p*-value.

**Table 3:** The distribution of study participants (%) for each PHQ-15 item.

PHQ-15	0—“It Does Not Bother Me at All”	1—“It Bothers Me a Little”	2—“It Bothers Me a Lot”	1 + 2
1. Back pain (%)	29.8	40.9	29.4	70.3
2. Thoracic pain in the chest area (%)	55.0	33.9	11.1	45.0
3. Constipation, loose bowels, or diarrhea (%)	52.7	33.9	13.4	47.3
4. Dizziness (%)	40.3	41.5	18.2	59.7
5. Fainting spells (syncope) (%)	91.9	6.5	1.6	8.1
6. Feeling tired or having low energy (%)	5.7	34.3	59.9	94.2
7. Feeling your heart pound or race (%)	49.1	30.8	20.0	50.8
8. Headaches (%)	26.7	47.8	25.6	73.4
9. Menstrual cramps ^a^ (%)	37.1	28.6	34.3	62.9
10. Nausea, gas, or indigestion (%)	43.9	33.9	22.2	56.1
11. Pain or problems during sexual intercourse (%)	80.9	13.2	5.9	19.1
12. Shortness of breath (%)	79.6	14.4	6.0	20.4
13. Stomach pain (%)	56.8	32.1	11.0	43.1
14. Trouble sleeping (%)	27.5	37.6	34.9	72.5
15. Pain in arms, legs, or joints (knees, hips, etc.)	82.1	10.6	7.3	17.9

^a^—The data of item 9 are restricted to the male sample.

**Table 4:** Body composition characteristics dependent on sex.

Variables	Males (*n* = 211)	Females (*n* = 1012)	Norm	*d*	*p*
Standing height (m)	1.8 ± 0.1	1.7 ± 0.1	—	1.7	<0.001
Body weight (kg)	78.2 ± 14.5	62.9 ± 11.3	—	1.1	<0.001
Body mass index (kg/m^2^)	23.2 ± 3.7	22.0 ± 3.6	18.5–25.0	0.3	<0.001
Body fat (kg)	14.6 ± 7.9	18.1 ± 7.5	—	−0.5	<0.001
Body fat (%)	17.6 ± 6.4	27.7 ± 6.1	≤20 in M and ≤30 in F	−1.4	<0.001
Fat-free mass (kg)	63.6 ± 7.5	44.9 ± 4.5	—	2.1	<0.001
Fat-free mass (%)	82.3 ± 6.4	72.3 ± 6.0	75–85% in M and 70–80% in F [86]	1.4	<0.001
Fat-free mass index (kg/m^2^)	18.8 ± 1.4	15.7 ± 1.1	22–23 in M and 18–19 in F	1.9	<0.001
Adjusted fat-free mass index (kg/m^2^)	18.7 ± 1.5	16.3 ± 1.2		1.5	<0.001

Data are presented as mean ± SD. *d*—the effect size; *p*—*p*-value; M—males; F—females.

**Table 5:** Association between body fat mass (%) and somatization as a dependent variable.

Model	Dependent Variable	β	95% CI [LB; UB]	*p*	F_5,1218_	*R* ^2^
1. BF% ^a^	PHQ-15 scale (total score)	0.050	[0.013; 0.084]	0.007	97.6	0.32
1.1. BF% ^a×1^	Back pain (score) ^1^	0.010	[0.001; 0.012]	0.051	13.0	0.26
1.2. BF% ^a×2^	Thoracic pain in chest area (score) ^2^	0.001	[−0.005; 0.005]	0.092	26.6	0.11
1.3. BF% ^a×3^	Constipation, loose bowels, or diarrhea (score) ^3^	0.010	[0.002; 0.013]	0.005	22.2	0.20
1.4. BF% ^a×4^	Dizziness (score) ^4^	0.001	[−0.005; 0.005]	0.995	39.9	0.16
1.5. BF% ^a×5^	Fainting spells (syncope) (score) ^5^	−0.001	[−0.003; 0.002]	0.564	5.7	0.03
1.6. BF% ^a×6^	Feeling tired or having low energy (score) ^6^	0.003	[−0.001; 0.007]	0.149	59.1	0.20
1.7. BF% ^a×7^	Feeling your heart pound or race (score) ^7^	−0.002	[−0.007; 0.004]	0.570	47.2	0.19
1.8. BF% ^a×8^	Headaches (score) ^8^	0.005	[0.001; 0.011]	0.050	24.9	0.21
1.9. BF% ^a×9^	Menstrual cramps (score) ^9^	0.022	[0.016; 0.028]	<0.001	21.4	0.24
1.10. BF% ^a×10^	Nausea, gas, or indigestion ^10^	0.007	[0.001; 0.013]	0.014	23.7	0.23
1.11. BF% ^a×11^	Pain or problems during sexual intercourse (score) ^11^	0.001	[−0.004; 0.004]	0.959	10.2	0.05
1.12. BF% ^a×12^	Shortness of breath (score) ^12^	0.002	[−0.002; 0.006]	0.411	21.2	0.09
1.13. BF% ^a×13^	Stomach pain (score) ^13^	0.001	[−0.005; 0.006]	0.862	22.7	0.10
1.14. BF% ^a×14^	Trouble sleeping (score) ^14^	0.001	[−0.006; 0.005]	0.815	40.0	0.16
1.15. BF% ^a×15^	Pain in arms, legs, or joints (knees, hips, etc.) (score) ^15^	−0.002	[−0.006; 0.002]	0.395	14.3	0.07

^a^—Dependent variable for multiple linear regression models. ^1–15^—independent variables (in terms of somatic symptoms of PHQ-15) for multiple linear regression models. All regression models were adjusted for confounders: sex and scores of the HADS-Anxiety scale, HADS-Depression scale, the sports index, and the work index. BF%—body fat percentage; *p*—*p*-value; 95% CI—95% confidence interval; LB—lower bound; UB—upper bound; F—the F-statistic; *R*^2^—the *R*-Squared.

**Table 6:** Association between fat-free mas index (kg/m^2^) and somatization as a dependent variable.

Model	Dependent Variable	β	95% CI [LB; UB]	*p*	F_5,1218_	*R* ^2^
1. FFMI_adj_ (kg/m^2^) ^a^	PHQ-15 scale (total score)	−0.429	[−0.597; −0.260]	<0.001	101.9	0.34
1.1. FFMI_adj_ (kg/m^2^) ^a×1^	Back pain (score) ^1^	−0.028	[−0.056; 0.001]	0.050	22.1	0.20
1.2. FFMI_adj_ (kg/m^2^) ^a×2^	Thoracic pain in chest area (score) ^2^	−0.027	[−0.052; −0.003]	0.028	27.5	0.26
1.3. FFMI_adj_ (kg/m^2^) ^a×3^	Constipation, loose bowels, or diarrhea (score) ^3^	−0.016	[−0.043; 0.010]	0.225	11.1	0.05
1.4. FFMI_adj_ (kg/m^2^) ^a×4^	Dizziness (score) ^4^	−0.057	[−0.082; −0.032]	<0.001	43.9	0.28
1.5. FFMI_adj_ (kg/m^2^) ^a×5^	Fainting spells (syncope) (score) ^5^	0.001	[−0.012; 0.014]	0.923	5.6	0.03
1.6. FFMI_adj_ (kg/m^2^) ^a×6^	Feeling tired or having low energy (score) ^6^	−0.022	[−0.042; −0.001]	0.036	59.6	0.25
1.7. FFMI_adj_ (kg/m^2^) ^a×7^	Feeling your heart pound or race (score) ^7^	−0.036	[−0.063; −0.009]	0.008	48.6	0.21
1.8. FFMI_adj_ (kg/m^2^) ^a×8^	Headaches (score) ^8^	−0.027	[−0.053; −0.001]	0.044	24.9	0.20
1.9. FFMI_adj_ (kg/m^2^) ^a×9^	Menstrual cramps (score) ^9^	−0.146	[−0.176; −0.116]	<0.001	28.2	0.22
1.10. FFMI_adj_ (kg/m^2^) ^a×10^	Nausea, gas, or indigestion ^10^	−0.034	[−0.062; −0.006]	0.018	23.6	0.24
1.11. FFMI_adj_ (kg/m^2^) ^a×11^	Pain or problems during sexual intercourse (score) ^11^	−0.020	[−0.040; 0.001]	0.058	10.9	0.05
1.12. FFMI_adj_ (kg/m^2^) ^a×12^	Shortness of breath (score) ^12^	0.013	[−0.007; 0.033]	0.211	21.4	0.09
1.13. FFMI_adj_ (kg/m^2^) ^a×13^	Stomach pain (score) ^13^	−0.019	[−0.044; 0.005]	0.125	23.1	0.11
1.14. FFMI_adj_ (kg/m^2^) ^a×14^	Trouble sleeping (score) ^14^	−0.016	[−0.044; 0.110]	0.241	40.3	0.16
1.15. FFMI_adj_ (kg/m^2^) ^a×15^	Pain in arms, legs, or joints (knees, hips, etc.) (score) ^15^	0.006	[−0.015; 0.027]	0.597	14.2	0.07

^a^—Dependent variable for multiple linear regression models. ^1–15^—independent variables (in terms of somatic symptoms of PHQ-15) for multiple linear regression models. All regression models were adjusted for confounders: sex and scores of the HADS-Anxiety scale, the HADS-Depression scale, the sports index, and the work index. FFMI_adj_—adjusted fat-free mass index; *p*—*p*-value; 95% CI—95% confidence interval; LB—lower bound; UB—upper bound; F—the F-statistic; *R*^2^—the *R*-Squared.

## Data Availability

The data are available on request.
